# Rapid Colorimetric Testing for Pyrazinamide Susceptibility of *M. tuberculosis* by a PCR-Based *In-Vitro* Synthesized Pyrazinamidase Method

**DOI:** 10.1371/journal.pone.0027654

**Published:** 2011-11-10

**Authors:** Man Zhou, Xuelei Geng, Jun Chen, Xude Wang, Dianbing Wang, Jiaoyu Deng, Zhiping Zhang, Weihua Wang, Xian-En Zhang, Hongping Wei

**Affiliations:** 1 State Key Laboratory of Virology, Wuhan Institute of Virology, Chinese Academy of Sciences, Wuhan, China; 2 Wuan Tuberculosis Control Center, Baofeng Road, Wuhan, China; 3 Graduate School, Chinese Academy of Sciences, Beijing, China; National Institute of Allergy and Infectious Disease, United States of America

## Abstract

Pyrazinamide (PZA) is an important first-line anti-tuberculosis drug. But PZA susceptibility test is challenging because PZA activity is optimal only in an acid environment that inhibits the growth of M. tuberculosis. For current phenotypic methods, inconsistent results between different labs have been reported. Direct sequencing of pncA gene is being considered as an accurate predictor for PZA susceptibility, but this approach needs expensive sequencers and a mutation database to report the results. An in-vitro synthesized Pyrazinamidase (PZase) assay was developed based on PCR amplification of pncA gene and an *in vitro* wheat germ system to express the pncA gene into PZase. The activity of the synthesized PZase was used as an indicator for PZA susceptibility. Fifty-one clinical isolates were tested along with pncA sequencing and the BACTEC MGIT 960 methods. The *in-vitro* synthesized PZase assay was able to detect PZA susceptibility of M. tuberculosis within 24 h through observing the color difference either by a spectrometer or naked eyes. This method showed agreements of 100% (33/33) and 88% (14/16) with the pncA sequencing method, and agreements of 96% (27/28) and 65% (15/23) with the BACTEC MGIT 960 method, for susceptible and resistant strains, respectively. The novel *in-vitro* synthesized PZase assay has significant advantages over current methods, such as its fast speed, simplicity, no need for expensive equipment, and the potentials of being a direct test, predicting resistance level and easy reading results by naked eyes. After confirmation by more clinical tests, this method may provide a radical change to the current PZA susceptibility assays.

## Introduction

The emergence of drug-resistant strains is one of the main causes for the current spread of Tuberculosis (TB) and it is clinically important to do drug susceptibility testing for better control of TB [1], [2]. Pyrazinamide (PZA) is an important first-line anti-tuberculosis drug used in combination with isoniazid, rifampicin and ethambutol in the short-course treatment regimens recommended by the WHO [3]. While the procedures for susceptibility testing of the first-line and second-line drugs have been well standardized in both liquid and solid media, the PZA susceptibility test is challenging because the bactericidal activity of PZA is optimal only in an acid environment (pH 5.5–5.6) that inhibits the growth of *M. tuberculosis* isolates [4], [5].

Many phenotypic methods [6], [7], [8], [9] were developed to perform the assay at higher pH (around 6.0) and increased PZA concentrations, which are far beyond the active PZA concentration *in vivo*. Among them, the radiometric assay (BACTEC 460TB) using pH 6.0 and PZA concentration of 100ug/mL was considered as most reliable and the currently recommended assay (Clinical Laboratory Standard Institute, M24-A2). However, even for this reference method, inconsistent results between different labs have been reported [10], [11]. Previous studies found that the radiometric assay can lead to inconclusive results up to 3.5% of the strains [12], falsely resistant results for at least 0.8% of the strains [13], and falsely susceptible results for some strains [14].

Direct sequencing of *pncA* gene is being considered as a more reliable and accurate method to predict PZA susceptibility [14], [15]. Since *pncA* gene (GenBank accession number U59967), encoding PZase, was cloned in 1996 [16], a lot of research confirmed that mutations in *pncA* could result in lost or reduced PZase activity, and such mutations were proven to be the primary mechanism of PZA resistance in *M. tuberculosis* [17], [18], [19]. The fact that over 90% of PZA-resistant *M. tuberculosis* isolates carry mutations in the coding region of *pncA,* makes it possible to test PZA susceptibility through monitoring mutations in the *pncA* gene [17], [20], [21]. Because PZA resistance mutations are highly diverse and found throughout the entire *pncA* gene, direct sequencing of PCR products and DNA microarrays [22] were used frequently. However, these methods are not commonly available due to costly apparatus, and could only be used for predication or need a pre-known database to link the mutation sites with the susceptibility results. For new mutation sites, further confirmation by phenotypic testing is needed.

A hybrid genetic and phenotypic method based on PCR and *in-vitro* cell free expression systems to do rapid PZase assay might be an ideal choice for PZA susceptibility test [23]. Due to the strong red color of the rabbit reticulocyte lysate used in the previous study, a tedious treatment was needed to remove the red hemoglobin to avoid the interference with PZase assay. More troublesome, two pairs of PCR primers were needed in order to cover all possible mutations in the *pncA* genes, which mean double PCR and *in-vitro* expression for one sample and therefore, significant increase of cost and work load. All these issues limited further applications of this method.

In this study, we developed a simple colorimetric method to enable rapid PZA susceptibility testing based on PCR amplification of *pncA* gene and an *in-vitro* wheat germ system. We also demonstrated, for the first time, that this method may have the potential of being a direct test for sputum specimens, therefore, bypassing the lengthy cultivation of tubercle bacilli.

## Results

### Amplification of *pncA* gene by primers with or without 5′-UTR sequence

**Figure 1:**
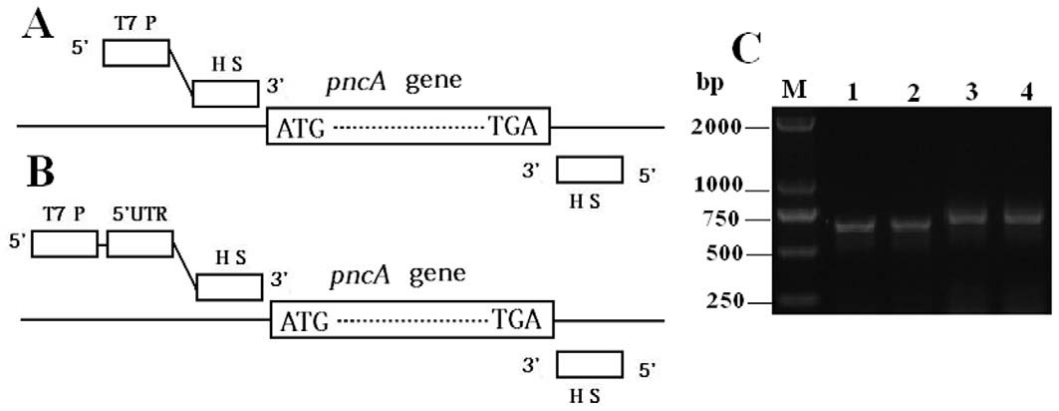
Schematic presentation of the primer positions without (A) and with 5′UTR sequence (B), as well as gel electrophoresis image of the PCR products (C). Lane M, markers; Lane 1, *M. tuberculosis* H37Ra template with primers F-NO5 and R-1; Lane 2, *M. bovis* BCG template with primers F-NO5 and R-1; Lane 3, *M. tuberculosis* H37Ra template with primers F-5 and R-1; Lane 4, *M. bovis* BCG template with primers F-5 and R-1. HS: hybridization sequence complement to genomic DNA of *M. tuberculosis*.

In order for PCR products to cover possible mutations on the whole *pncA* gene, PCR primers were designed from the upstream and the downstream regions of *pncA* gene on *M. tuberculosis* genome (Fig. 1A and 1B). A T7 promoter was attached with the forward primers in order for the wheat germ system to express protein directly from PCR products. To shorten *in-vitro* expression time, 5′-untranslated regulatory regions (5′UTR) were also considered since some 5′UTR sequences could enhance protein expression in wheat germ system [24]. After optimization, two pairs of primers shown in the supplementary Table S1 were synthesized and used to amplify *pncA* gene. As shown in Fig. 1C, both primer pairs could effectively amplify *pncA* gene of *M. tuberculosis* H37Ra and *M. bovis* BCG. Similar electrophoresis results were also obtained for *pncA* genes amplified from all the clinical samples tested (data not shown).

### Optimization of primers and incubation time for *in-vitro* synthesis of PZase

**Figure 2:**
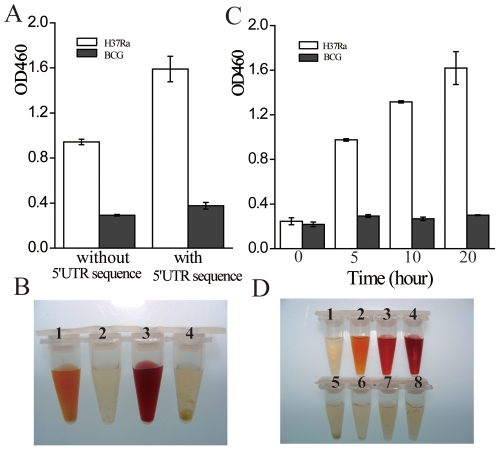
Activities of *in-vitro* synthesized PZase. Templates used for the PCR were *M. tuberculosis M. tuberculosis* H37Ra (PZA susceptible; white bar) and *M. bovis* BCG (PZA resistant; black bar). Optical density (A) and colors (B) of PZase activities obtained by using PCR products from *M. tuberculosis* H37Ra and *M. bovis* BCG with (F-5 as the forward primer, tube 3: *M. tuberculosis* H37Ra; tube 4: *M. bovis* BCG) and without (F-NO5 as the forward primer, tube 1: *M. tuberculosis* H37Ra; tube 2: *M. bovis* BCG) the 5′UTR sequence. Incubation time for *in-vitro* synthesis: 20 h. (C) Time course study of the synthesized PZase activities with F-5 as the forward primer and their corresponding colors (D). Tubes 1–4: *M. tuberculosis* H37Ra, incubation for 0, 5, 10 and 20 hours, respectively. Tubes 5–8: *M. bovis* BCG, incubation for 0, 5, 10 and 20 hours, respectively.

Fig. 2A and 2B show a comparison of PZase activities of *M. tuberculosis* H37Ra and *M. bovis* BCG obtained by using the primer pairs with or without the 5′UTR sequence, respectively. Using F-5 and R-1 as the primers, the PZase activity obtained with the *pncA* gene amplified from *M. tuberculosis* H37Ra was about double (tube 3, Fig. 2B) to that using F-NO5 and R-1 as the primers (tube 1, Fig. 2B). While minimum PZase activity (no red color) was observed with the *pncA* gene amplified from *M. bovis* BCG using either primer pairs (tubes 2 and 4, Fig. 2B). Thus, the *in-vitro* PZase synthesis was more efficient using the PCR products that contain the 5′UTR sequence. Fig. 2C shows that the PZase activity of the lysate containing the *M. tuberculosis* H37Ra PCR product increased continuously in a time-dependent manner, which was clearly due to that more PZase was synthesized in longer reaction time. In contrast, almost no PZase activity was observed in the lysates containing the *M. bovis* BCG PCR product even after 20 hours of incubation. An incubation time of 10 hours was chosen in terms of speed and efficiency. In the following experiments, primers F-5 and R-1 were used as the PCR primers and the expression time was set at 10 hours.

### PZA susceptibility testing of *M. tuberculosis* clinical samples by the *in-vitro* synthesized PZase assay

PZA susceptibility of 51 clinical isolates was examined. In order to know the reproducibility and obtain the cut-off threshold to judge PZA susceptibility, the 51 isolates were divided into three batches and tested together with *M. tuberculosis* H37Ra and *M. bovis* BCG. In each batch, each sample was tested twice; *M. tuberculosis* H37Ra and *M. bovis* BCG were tested in three repeats. The samples were processed and the optical density (OD) ratios of the samples including *M. tuberculosis* H37Ra and *M. bovis* BCG were calculated by dividing the OD value of each well to the mean OD value from three replicate *M. tuberculosis* H37Ra tubes in the same batch. Because any mutations resulting reduced PZase activity would generate PZA resistance, a clinical sample was scored as resistant if the mean OD ratio from its two replicate tubes was lower than a cutoff threshold, which was set at the mean minus three SDs of the OD ratios of all the nine tubes of *M. tuberculosis* H37Ra in the three batches.

**Figure 3:**
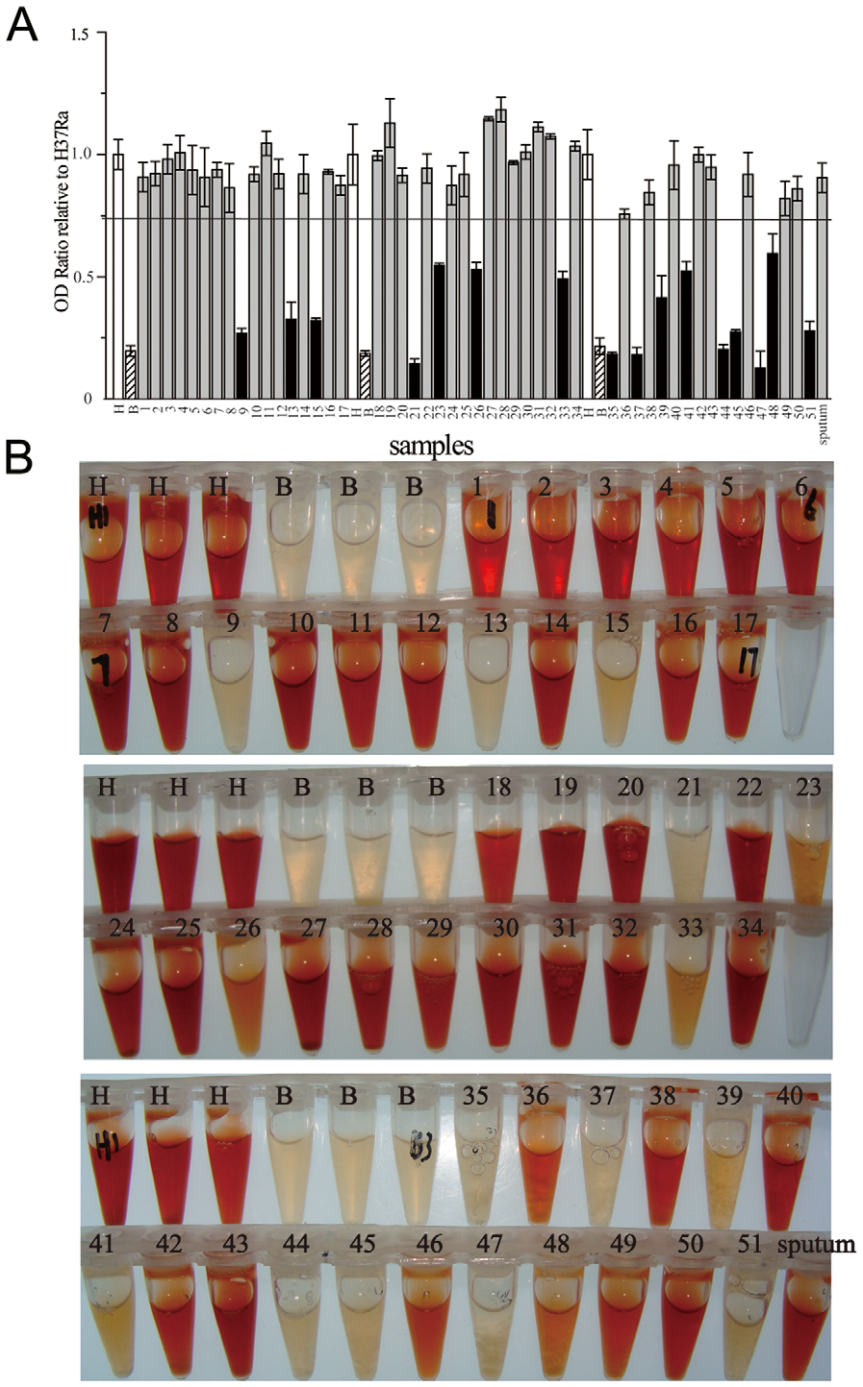
*In-vitro* synthesized PZase activities of clinical samples. A. OD ratios of samples relative to *M. tuberculosis* H37Ra. The samples with their OD ratios relative to *M. tuberculosis* H37Ra below the cutoff were marked as black bars (PZA resistant). The samples with their OD ratios relative to *M. tuberculosis* H37Ra above the cutoff were marked as gray bars (PZA susceptible). H: *M. tuberculosis* H37Ra, white bars; B: *M. bovis* BCG, hatched bars.Sputum: sputum sample. Clinical isolates are labeled with their specimen No. **B**. Pictures of *in-vitro* synthesized PZase activities of all the clinical samples. H: *M. tuberculosis* H37Ra; B: *M. bovis* BCG. Sputum: sputum sample. Clinical isolates are labeled with their specimen No.

Fig. 3A and 3B show OD ratios relative to *M. tuberculosis* H37Ra and the pictures of the *in-vitro* synthesized PZase activities of all the samples, respectively. Among all the samples tested, 16 samples exhibited PZase activities with their OD ratios relative to *M. tuberculosis* H37Ra lower than the cutoff. These 16 samples were considered as PZA resistant. The remaining 35 samples were considered as PZA susceptible with their OD ratios relative to *M. tuberculosis* H37Ra higher than the cutoff. From Fig. 3B, it can be seen that the red color differences between PZA susceptible and resistant strains were so obvious that they could be easily discriminated by naked-eyes.

Sputum specimens with positive smear for AFB were also tested and only one typical result was shown in Fig. 3. Based on the Blast results, the primer sequences used in this study were quite specific to the genome of *M. tuberculosis*, and had the possibility for direct sputum tests. Generally we found that the success of this method for sputum specimens depended on the PCR results. Various PCR results were obtained for different sputum specimens (data not shown). We are still optimizing the DNA extraction process to establish the level of sensitivity (required minimal number of bacilli in the specimen).

### DNA sequencing and BACTEC MGIT 960 results of clinical isolates

**Figure 4:**
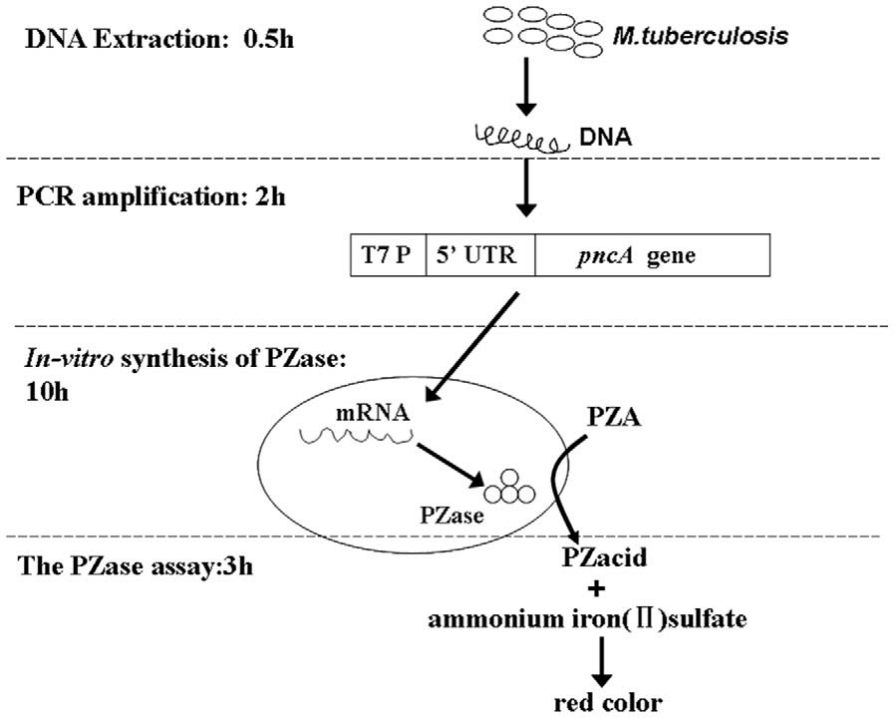
General procedure of the PCR-based *in-vitro* synthesized PZase assay. The *in-vitro* PZase assay consisted of four steps. (A) Extraction of DNA from samples. (B) PCR amplification of the *pncA* gene. (C) *In-vitro* synthesis of PZase. (D) Assay of PZase activity. T7 P: T7 promoter. 5′UTR: 5′untranslated regulatory regions containing translational enhancer elements.

The BACTEC results of the isolates and the sequencing results of *pncA* genes from the 51 isolates and the sputum specimen are presented in Table 1. Comparing these results with that of the *in-vitro* synthesized PZase assay, 27 isolates were found susceptible by the PZase assay (Fig. 4A) and the BACTEC test, and their *pncA* sequences revealed no mutations or a silent mutation (No. 17). These 27 samples were identified as PZA susceptible by all the three methods. 13 isolates were found resistant by the PZase assay and the BACTEC test, and their *pncA* sequences revealed that they have mutations, which were proved to lead to PZA resistance in previous studies [17], [18], [20], [21], [25]. These 13 isolates were identified as PZA resistant by all the three methods. In the remaining isolates, six isolates were identified as PZA susceptible by the PZase assay and the sequencing results, but PZA resistant by the BACTEC test. Isolates No. 24 and 49 showed no loss of PZase activity, which indicated PZA susceptible by the *in-vitro* PZase assay. But the sequencing results revealed that No. 24 had a mutation C(512)→A and No. 49 had two mutations C(458)→T and C(474)→A. These site mutations were not reported before whether lead to PZA resistance or not. The BACTEC test showed these two isolates were resistant. For the two isolates (No. 26 and 44), whose DNA sequences could not been given due to the double sequencing peaks (Figure S1), both the *in-vitro* PZase assay and the BACTEC results showed PZA resistant. The sputum sample was identified as PZA susceptible by the PZase assay and its sequencing result.

**Table 1:** Results of PZA susceptibility of *M. tuberculosis* clinical samples tested by *pncA* sequencing, *in*-*vitro* synthesized PZase assay and BACTEC MGIT 960 System.

No. of Strains	*pncA* sequencing results and mutations	PZase mutations	PZA susceptibility by sequencing	PZA susceptibility by the *in-vitro* PZase assay	PZA susceptibility by BACTEC 960
1,2,3,4,5,6,7,8,11, 14,16,19,20,22,2527,28,29,30,3132, 36, 40,42,43,46,Sputum sample^d^	WT^a^	WT^a^	S	S	S
10,12,18,34,38,50	WT^a^	WT^a^	S	S	R
9	T(202)→C	Trp (86)→Arg	R ref. [20]	R	R
13	G (362)→C	Arg(121)→Pro	R ref. [21]	R	R
15	C (169)→T	His(57)→Tyr	R ref. [2]	R	R
17	C (234)→T	WT^b^	S	S	S
21	A (29)→C	Gln(10)→Pro	R ref. [18]	R	R
23	A insertion between nt 192 and 193	Frame shift	R	R	R
24	C(512)→A	Ala(171)→Glu	NA	S	R
26	——c	——c	NA	R	R
33	T(416)→G	Val(139)→Gly	R ref. [20]	R	R
35	G(145)→T	Asp(49)→Tyr	R	R	R
37	A(188)→C	Asp(63)→Ala	R ref. [28]	R	S
39	T(416)→G	Val(139)→Gly	R ref. [20]	R	R
41	T(199)→C	Ser(67)→Pro	R ref. [20]	R	R
44	——c	——c	NA	R	R
45	C(169)→T	His(57)→Tyr	R ref. [2]	R	R
47	T insertion between nt 396 and 397	Frame shift	R	R	R
48	T(515)→C	Leu(172)→Pro	R ref. [2]	R	R
49	C(458)→T, C(474)→A	Thr(153)→Ile,Asp(159)→Glu	NA	S	R
51	57–59, 62–65, 70–74 deleted	Frame shift	R	R	R

a , wild type sequence.

b , silent mutation, the amino acid sequence was not changed.

c , sequencing result is disordered.

d , sputum sample was not tested by the BACTEC MGIT 960 system.

S, PZA susceptible.

R, PZA resistant.

NA, not available.

ref. , reference that reported the mutation sites leading to PZA resistance.

As shown in Table 2, overall the *in-vitro* synthesized PZase results showed agreements of 100% (33/33) and 88% (14/16) with the *pncA* sequencing results, and agreements of 96% (27/28) and 65% (15/23) with the BACTEC MGIT 960 results, for susceptible and resistant strains, respectively.

**Table 2:** Comparison of the *in-vitro* synthesized PZase assay with BACTEC MGIT 960 and *pncA* sequencing.

N = 51	BACTEC MGIT 960		*pncA* sequencing		
	Susceptible (28)	Resistant (23)	Wild type/silent mutant (33)	Mutants (16)	Noresult(2)
**In-vitro PZase** Susceptible (35)	27	8	33	2	0
**In-vitro PZase** Resistant (16)	1	15	0	14	2
**Sensitivity**	65%		88%		
**Specificity**	96%		100%		

## Discussion

The *in-vitro* PZase method showed very good agreements (100% and 88% for susceptible and resistant strains, respectively) in results with the *pncA* sequencing method, which demonstrated that this method could be used to replace the *pncA* sequencing method for accurate prediction of PZA susceptibility. Compared with the direct sequencing method, the *in-vitro* synthesized PZase assay provides a ‘yes/no’ PZA resistance by measuring if the activity of the synthesized PZase is reduced related to that of the wild type PZase, which is easy and cheap to perform in most clinical labs without the need of expensive DNA sequencers. Furthermore, the *in-vitro* PZase method has a significant advantage that it does not need a pre-known mutation database to report PZA susceptibility results. As shown in Table 1, this method can give the susceptibility results for all the isolates, including those with no available linkage of their *pncA* mutation sites to resistance (isolates 24 and 49) and those that their *pncA* sequences could not be obtained due to the double peak problem (isolates 26 and 44, Figure S1). This advantage would be especially important for clinical detections because new mutations are expected to come out consistently for *M. tuberculosis* under pressure of PZA.

Compared with the BACTEC MGIT 960 method, the *in-vitro* PZase method is much faster (within 1 day) and safer for testing *M. tuberculosis* isolates since it does not need to grow the cells, which would eliminate the requirement of bio-safety level 3 lab for TB diagnostics and remove one major stumbling block to expanding lab capacity. Although these two methods showed good agreement (96%) for PZA susceptible strains, only moderate agreement (65%) for PZA resistant strains was obtained. The moderate agreement may be partly due to that there are other PZA resistant mechanisms other than mutations in *pncA* gene, but most probably due to false resistance of the Bactec test with 100 ug/ml PZA concentration as the breakpoint [18]. As shown in Table 1, there were six isolates recognized as PZA resistant by the Bactec test, but did not have any mutation in their gene sequences. As suggested before [5], [26], these six isolates might not be resistant to PZA at all. Without considering these 6 isolates, the agreement would be 88% (15/17) for PZA resistant strains.

Besides the advantages mentioned above, the *in-vitro* synthesized PZase assay also provides some unique potentials which may change the current PZA susceptibility assays. First, the incorporation of the 5′-UTR sequence into the PCR products effectively enhanced the PZase expression, which made it easy to differentiate the PZA resistant and susceptible samples in short time. As shown in Fig. 3, the color difference was so obvious that it was possible to use the color of *M. tuberculosis* H37Ra as the susceptible control and the color of *M. bovis* BCG as the resistant control to aid naked eyes to judge the susceptibility results. Secondly, initial studies showed that this method could be used as a direct test for sputum specimens, which would reduce the total turnaround time to 1–2 days without the time-costly isolation of *M. tuberculosis*. Finally, the PZase activity of each sample may be used to predict its resistance level, which may be more useful and more powerful evidence than currently exists to support any culture-based breakpoints. One recent study of recombinant mutated PZases showed that the level of resistance was significantly associated with Pzase activity [27]. As shown in Fig. 3, the *in-vitro* synthesized PZase of 16 resistant isolates had different activities. From their corresponding mutation sites revealed by the sequencing, it could find that the mutations causing significant loss of PZase activity (such as isolates 9, 15, 35 and 45, with color close to that of *M. bovis* BCG) are those that alter the catalytic residues comprising the active sites (D8, K96, A134 and C138) and the iron-binding sites (D49, H51, H57 and H71) revealed by the crystal structure of PZase [28]. Mutations located farther away (such as isolates 24 and 49, with color close to that of *M. tuberculosis* H37Ra) have less or no effect on PZase activity. These results suggest that the PZase activity measured in this method correctly reflected the changes of PZase due to mutations, which change its ability to convert PZA, i.e., the resistance level. However, all these potentials need further studies to confirm.

To conclude, initial studies showed that the new PCR-based *in-vitro* synthesized PZase assay had better performance than the sequencing method in determining PZA susceptibility of *M. tuberculosis* isolates and may have the potential to replace the BACTEC MGIT 960, which is considered as a safer alternative to the current golden standard radiometric BACTEC 460TB, for PZA testing. This method showed some significant advantages over current methods, such as its fast speed, simplicity and potentials of being a direct test and easy reading results by naked eyes. However, more clinical testing is needed to further evaluate the method and more studies are needed to confirm its potentials. If all these are going well, the *in-vitro* synthesized PZase assay may provide a revolutionary PZA susceptibility assay for the clinical labs and help to change the situation that most hospitals in developing countries do not test PZA susceptibility.

## Materials and Methods

### Bacterial strains

*M. tuberculosis* strain *M. tuberculosis* H37Ra (PZA susceptible) and *M. bovis M. bovis* BCG strain Tokyo (PZA resistant) have been maintained in our laboratory. All the clinical isolates and the sputum samples with positive smear for acid-fast bacilli (AFB) used in this study were derived from the sputum of patients treated in Wuhan Tuberculosis Control Center (Wuhan, China) and chosen randomly. The *M. tuberculosis* isolate cells were collected from the standard Lowenstein-Jensen media seeded with the sputum samples. Before the susceptibility test, the isolates were stored at a 4°C refrigerator. Written informed consent was obtained from all participants. The study and the consent procedure were approved by the Ethical and Scientific Committee of Wuhan Tuberculosis Control Center and the Academic and Ethical Committee of Wuhan Institute of Virology, Chinese Academy of Sciences.

### General procedure of the *In-Vitro* Synthesized Pyrazinamidase method

As shown in Fig. 4, the main steps consisted of PCR amplification of the *pncA* genes extracted from clinical samples, *in-vitro* expression of the PCR products and determination of the activities of the synthesized PZase. The total turnaround time was less than 24 h. The detailed experimental conditions are given in the supporting information sections (Method S1). Briefly, tubercle bacilli were collected from L–J medium (for isolates) or sputum specimens, and inactivated at 100°C for 10 min to obtain the DNA templates. Then, *pncA* gene fragments were amplified by PCR with primers shown in Fig. 1 and Table S1. After that, the amplified *pncA* DNA were concentrated and used directly for *in-vitro* synthesis of PZase by RTS 100 Wheat germ CECF kits (5 Prime Inc., Gaithersburg, USA). Finally, the resulting lysates were used directly for PZase activity assay based on the color developed due to the reaction of iron (II) with pyrazinoic acid, which was converted from PZA by the *in-vitro* synthesized PZase. A sample was considered as resistant if its synthesized PZase showed any reduction in activity/color absorption compared with that of the PZase from *M. tuberculosis* H37Ra, otherwise, as susceptible.

### DNA sequencing

The PCR products (with primers F-5 and R-1) of the *pncA* genes (GenBank accession number U59967) from the isolates and the sputum samples were directly sequenced using the sequencing primer 5′-TAATACGACTCACTATAG-3′ by Invitrogen Inc. (Shanghai, China). The resulting sequences were compared with the *pncA* sequence of *M. tuberculosis* H37Ra to identify mutations associated with PZA resistance.

### Test PZA susceptibility by BACTEC MGIT 960

PZA susceptibility tests of the clinical isolates were performed on a **BACTEC MGIT 960** system using the PZA susceptibility test kits (Becton Dickinson, Sparks, MD) according to the manufacturer's instructions. A PZA concentration of 100 µg/ml (recommended breakpoint) was used.

## Supporting Information

Figure S1 The sequencing peaks of the *pncA* gene of *M. tuberculosis* isolates No 26 (A) and No 44 (B). The double peaks start from position 223 for the isolate No 26 and position 315 for the isolate No 44, respectively.
(TIF)

Table S1 Primer sequences used for amplification of the *pncA* gene of *M. tuberculosis.*
(DOC)

Method S1 Detailed conditions for the *In-Vitro* Synthesized Pyrazinamidase method.
(DOC)
