# Supplementary figures and images for: Rapid Colorimetric Testing for Pyrazinamide Susceptibility of M. tuberculosis by a PCR-Based In-Vitro Synthesized Pyrazinamidase Method
Source: PLoS One. 2011 Nov 10;6(11):e27654. doi: 10.1371/journal.pone.0027654 (PMC3213173; doi:10.1371/journal.pone.0027654)

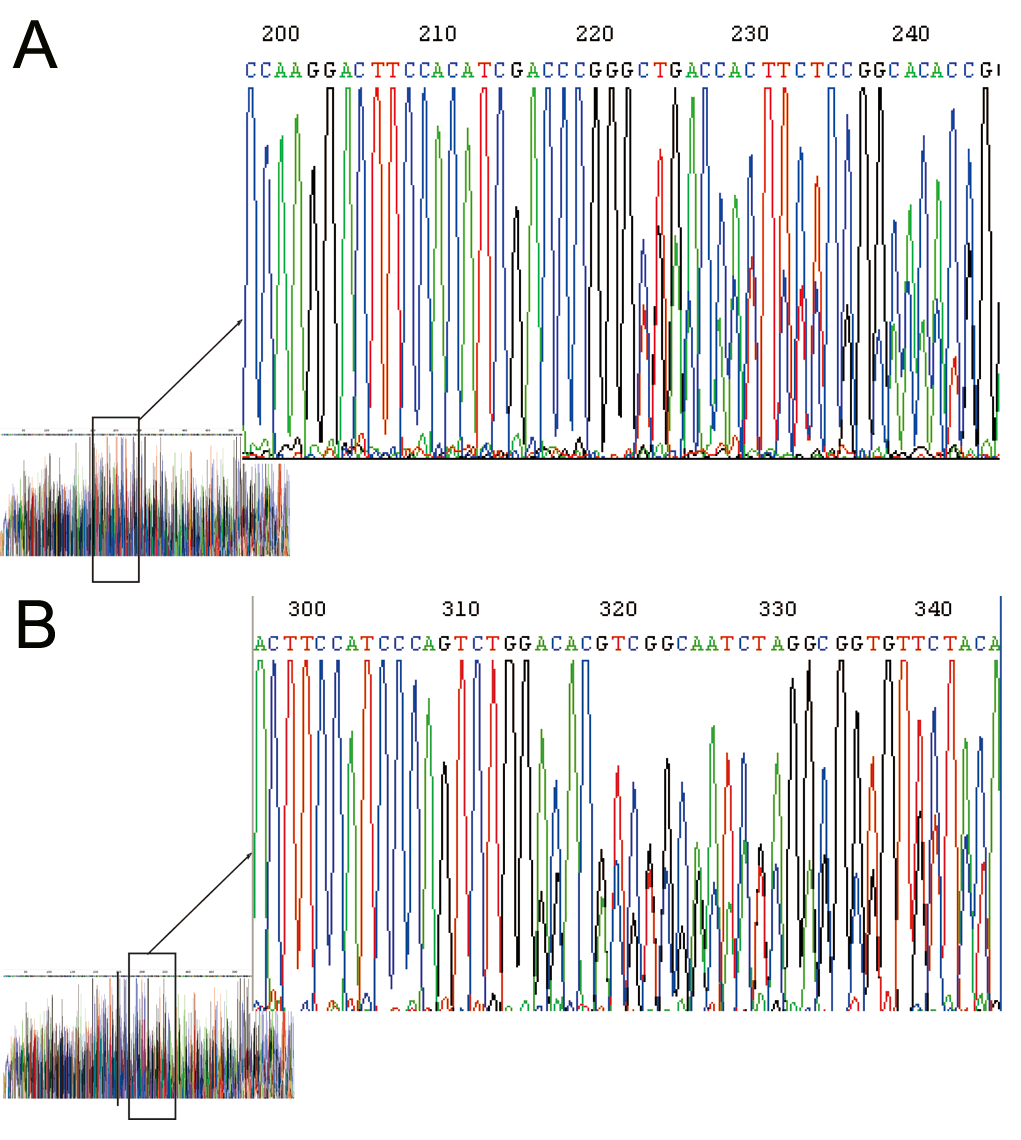

Supplement: Figure S1 — The sequencing peaks of the pncA gene of M. tuberculosis isolates No 26 (A) and No 44 (B). The double peaks start from position 223 for the isolate No 26 and position 315 for the isolate No 44, respectively. (TIF) [file pone.0027654.s001.tif]
