# Supplementary material for: Rapid Colorimetric Testing for Pyrazinamide Susceptibility of M. tuberculosis by a PCR-Based In-Vitro Synthesized Pyrazinamidase Method
Source: PLoS One. 2011 Nov 10;6(11):e27654. doi: 10.1371/journal.pone.0027654 (PMC3213173; doi:10.1371/journal.pone.0027654)
Supplement: Table S1 — Primer sequences used for amplification of the pncA gene of M. tuberculosis. (DOC) [file pone.0027654.s002.doc]

**Table S1.** Primer sequences used for amplification of the *pncA* gene of *M. tuberculosis.*

| Primer | Sequence *^a^* |
| --- | --- |
| F-NO5 | 5’ TAATACGACTCACTATAGGAGTCGCCCGAACGTAAGGAGGACGT3' |
| F-5^a^ | 5'TAATACGACTCACTATAGG**ATACTCCCCCACAACAGCTTACAATACTCCCCCACACAGCTTACAAATACTCCCCC**AGTCGCCCGAACGTAAGGAGGACGT3' |
| R-1 | 5'ACCGCCGCCAACAGTTCATCCCGGT3' |

*a,* the 5’UTR sequence is shown in boldface type.
