# Supplementary material for: Rapid Colorimetric Testing for Pyrazinamide Susceptibility of M. tuberculosis by a PCR-Based In-Vitro Synthesized Pyrazinamidase Method
Source: PLoS One. 2011 Nov 10;6(11):e27654. doi: 10.1371/journal.pone.0027654 (PMC3213173; doi:10.1371/journal.pone.0027654)
Supplement: Method S1 — Detailed conditions for the In-Vitro Synthesized Pyrazinamidase method. (DOC) [file pone.0027654.s003.doc]

**Method S1: Detailed conditions for the *In-Vitro* Synthesized Pyrazinamidase method**

**DNA template preparation**

Mycobacterium cells were collected from Lowenstein-Jensen medium. A loop of cells was suspended in 0.5 ml distilled water, and inactivated at 100℃ for 10 min. Cellular debris was pelleted at 13,000 rpm for 5 min, and the supernatant with the genomic DNA was used for PCR.

A sputum sample was collected from a patient who was suspected of having tuberculosis based on clinical symptoms and positive sputum smear for acid-fast bacilli (AFB). After treatment with 1 M NaOH, the sample was shaken for 10min. and then centrifuged at 13,000 rpm for 15min. The precipitate was washed twice with distilled water and mixed with 50μl of 0.1% TritonX-100. The mixture was inactivated at 100 ℃ for 10 min. Finally, the mixture was centrifuged at 15,000 rpm for 10 min, and the supernatant with the genomic DNA was used for PCR.

**Amplification of *pncA* gene.**

The *pncA* gene fragments were amplified by PCR with primers shown in Table S1. Amplification was performed in 0.2-ml PCR tubes with a total reaction volume of 50 μl by using a thermal cycler (Biometra, Germany). Each reaction tubes contained 0.6 U of Phusion®High-Fidelity DNA Polymerase (NEB, UK), 5 μl 10×PCR buffer, 200 μM of each dNTP, 20 μM of each primers, 4 μl Dimethyl-sulfoxide and 4 μl DNA template. PCR amplifications were conducted with an initial 30 s denaturation step at 98 ℃, 37 cycles of 98 ℃ for 30 s, 66 ℃ for 1 min, and 72 ℃ for 30 s, and a final 10 min elongation. The amplified products were analyzed by electrophoresis in 1.5% agarose gel with ethidium bromide.

**DNA concentration and quantization**

The amplified *pncA* DNA were concentrated by adding 3 M CH_3_COONa (pH 5.2) according to a volume ratio 1:10 and 4 μl DNAmate (Takara Biotechnology Co. Ltd, Dalian, China). Then 2.5 times volume of ethanol at – 20 ℃ was added and the mixtures were centrifuged at 12000 rpm for 15 min. Finally, the precipitate was dissolved in a volume of nuclease-free water after washing with 70% ethanol.

A nanodrop 2000 scientific spectrophotometer (Thermo Scientific Inc, USA) was used to evaluate the quantity and quality of the concentrated DNA. The DNA used for the *in-vitro* Wheat germ system was with concentrations between 800-1000 ng/μl and the ratios of 260nm/280nm between 1.70 and 1.85.

***In-vitro* synthesis of PZase from PCR products**

RTS 100 Wheat germ CECF kits were purchased from 5 Prime Inc. (Gaithersburg, USA) and reactions were carried out by following the procedures suggested by the manufacturer. The reaction volume was kept at 50 μl consisted of the following amounts of reagents: 15 μl wheat germ lysate, 15 μl reaction buffer, 4 μl amino acids, 1 μl methionine, and 8 μg PCR products. The feed solution was kept at 1 ml and prepared by mixing 900 μl feeding mix with 80 μl amino acids and 20 μl methionine. The protein expressions were conducted in an Eppendorf Thermomixer Comfort at 24 ℃ with 900 rpm of shaking. The resulting lysates were used directly for PZase assay, individually.

**PZase assay**

Measurements of PZase activities were performed with some modifications of the method described by McClatchy et al.[^25^](#_ENREF_25) After *in-vitro* synthesis of PZase, 20 μl of the lysates were mixed with 150 μl of 0.01 g/ml PZA in distilled water and incubated for 3 hours at 37 ℃, respectively. After incubation, 10 μl of 10% ammonium iron (Ⅱ) sulfate was added into the reaction mixtures. Red color would appear immediately for PZA sensitive samples. For measuring the absorbance at 460 nm, the mixtures were centrifuged at 10,000 rpm for 1min to remove possible particles in the lysate and the absorbance of the supernatants were measured by a Perkin Elmer Lambda 25 UV–Visible spectrophotometer.
